# What factors are associated with current smokers using or stopping e-cigarette use?

**DOI:** 10.1016/j.drugalcdep.2017.01.002

**Published:** 2017-04-01

**Authors:** Erikas Simonavicius, Ann McNeill, Deborah Arnott, Leonie S. Brose

**Affiliations:** a Department of Addictions, Institute of Psychiatry, Psychology and Neuroscience, King’s College London, London, United Kingdom; b Action on Smoking and Health, London, United Kingdom

**Keywords:** Electronic cigarettes, Smoking, Motivation to quit

## Abstract

**Background:**

While some smokers use e-cigarettes and stop smoking, a substantial proportion try e-cigarettes and later discontinue or use them concurrently with smoking (current dual users). The aim was to assess factors associated with ongoing e-cigarette use and discontinuation among smokers.

**Methods:**

Secondary analysis of data of 1489 currently smoking adults, 18 and above, from a GB population-based online survey conducted in March 2016. A multivariable logistic regression assessed motivation to stop smoking among never e-cigarette users, past triers (<weekly use), past users (≥weekly use), and current dual users. A multinomial logistic regression assessed associations of e-cigarette use status with motivation to stop smoking, reasons for use, age, gender, social grade, heaviness of smoking, and type of first e-cigarette used. Reasons for discontinuing use were assessed.

**Results:**

Current dual users were more motivated to stop smoking than past users (AOR = 1.95, 95% CI: 1.10-3.46); never users’ or past triers’ motivation did not differ from past users’. Dual users were less dependent on cigarettes (AOR = 0.54, 95% CI: 0.35–0.86) and more likely to use e-cigarettes as an aid to reduce smoking (AOR = 2.40, 95% CI: 1.59–3.64) and to deal with smoking restrictions (AOR = 2.03, 95% CI: 1.22–3.38) than past users. Smokers mostly discontinued e-cigarettes because they did not feel like smoking, did not help with cravings, or respondents had just wanted to try them.

**Conclusion:**

Among smokers, ongoing use of e-cigarettes is associated with reasons for reducing smoking and dealing with smoking restrictions, heightened motivation to stop smoking, and lower dependence on smoking.

## Highlights

• Some smokers also use e-cigarettes; many stop e-cigarette use but continue to smoke.
• Smokers using e-cigarettes are more motivated to stop smoking than other smokers.
• Smokers who discontinue using e-cigarettes have a higher dependence on smoking.

## 1 Introduction

The use of electronic cigarettes (‘vaping’) has risen substantially in recent years. In Great Britain, the number of adult users quadrupled from 700,000 in 2012 to 2.8 million in 2016 (6% of the adult population) (ASH, 2016a). Frequently reported reasons for use are to reduce or stop smoking (Amato et al., 2016, Biener and Hargraves, 2015, McNeill et al., 2015, Pepper et al., 2014), and in England, smokers more commonly use e-cigarettes than licensed nicotine replacement therapy (NRT) when trying to stop smoking (West and Brown, 2015). Similar proportions of e-cigarette and NRT users concurrently smoke (West et al., 2016); NRT use alongside smoking is associated with reduction in cigarettes smoked and increased chances of subsequently trying to stop (Beard et al., 2013, National Institute for Health and Care Excellence, 2013, Stead and Lancaster, 2007, Stead et al., 2008).

E-cigarettes are generally not medicinally licensed. However, the UK regulatory agency recognises that nicotine is effective in smoking cessation, therefore, if e-cigarettes show bio-equivalence to a licensed NRT product, they could be classified as medication (MHRA, 2013), as an electronic inhaler was in 2016 (MHRA, 2015). Regardless of medicinal licensing, it is agreed that continuing to smoke is more harmful to health than switching to e-cigarettes (Grana et al., 2014, McNeill et al., 2015; Royal College of Physicians, 2016). Therefore, the two thirds of smokers who try but later stop using e-cigarettes while continuing to smoke (ASH, 2016a, Manzoli et al., 2016, Pepper et al., 2014) constitute a missed opportunity for harm reduction or smoking cessation.

A few US surveys found that smokers’ main reasons for not continuing e-cigarette use were that e-cigarettes did not feel like tobacco cigarettes (Biener and Hargraves, 2015), were too expensive (Biener et al., 2015), or ‘just experimenting’ (Pepper et al., 2014). However, there is little information about which users are at risk of reverting to smoking only. Several studies have found higher motivation to stop smoking in e-cigarette users compared with non-users (Biener and Hargraves, 2015, Hitchman et al., 2015, Wagener et al., 2014), but there is no evidence on motivation to stop in former e-cigarette users who continue to smoke.

Therefore, we aimed to address two research questions:

1. What is the association between motivation to stop smoking and e-cigarette use status?
2. What reasons and characteristics are associated with smokers using and discontinuing e-cigarettes?

## 2 Methods

### 2.1 Design and sample

The study is a secondary analysis of a national online survey (ASH, 2016a). From a total of 12,157 respondents, never-smokers (*n* *=* 6090*;* 50.1%) and former smokers (*n* *=* 4367*;* 35.9%) were excluded. Smokers who had not heard of e-cigarettes or were unsure whether they had (*n* = 64; 3.8%), or did not report their heaviness of smoking (*n* = 147; 8.6%) were excluded, leaving 1489 smokers to address research question 1. To address research question 2, smokers who had never tried e-cigarettes (*n* *=* 536) were excluded, leaving *n* = 953.

### 2.2 Measures

#### 2.2.1 Sociodemographics

Respondents’ gender (male, female), age (18‐24, 25‐34, 35‐44, 45‐54, 55 + ), and social grade (National Readership Survey, 2016), dichotomized to ABC1 and C2DE, were recorded.

#### 2.2.2 Smoking

Smoking status was assessed using ‘Smoking in this survey refers to all burnt tobacco products. It does NOT include e-cigarettes. Which of the following statements BEST applies to you?’ I smoke but I don't smoke every day; I smoke every day; I used to smoke but have given up now (excluded); I have never smoked (excluded). Dependence was measured using the Heaviness of Smoking Index (HSI) (Heatherton et al., 1989), dichotomised to ‘Low dependence’ (0–3) and ‘High dependence’ (4–6) (Diaz et al., 2005).

The Motivation to Stop Scale (MTSS) is a single item predictive of subsequent smoking cessation attempts (Hummel et al., 2016, Kotz et al., 2013). Responses were dichotomized (Hitchman et al., 2015), differentiating smokers motivated to stop in the next one or three months from others.

#### 2.2.3 E-cigarette use

E-cigarette use status was assessed using ‘Which of the following statements BEST applies to you?’ I have never heard of e-cigarettes and have never tried them (excluded); I have heard of e-cigarettes but have never tried them (‘never users’); I have tried e-cigarettes but do not use them (anymore) (‘past e-cigarette experience’); I have tried e-cigarettes and still use them (‘current dual users’); Don’t know (excluded). Those with past e-cigarette experience were divided by frequency of use; ‘past triers’ had only tried once or twice or used less than twice a month and ‘past users’ had used at least once a week (Amato et al., 2016).

Type of first e-cigarette used was chosen from: Disposable electronic-cigarette (non-rechargeable); Electronic cigarette kit that is rechargeable with replaceable pre-filled cartridges; Electronic cigarette that is rechargeable and has a tank or reservoir that you fill with liquids; Other; Don’t know/can’t remember.

#### 2.2.4 Reasons for using and discontinuing use

**Fig. 1:**
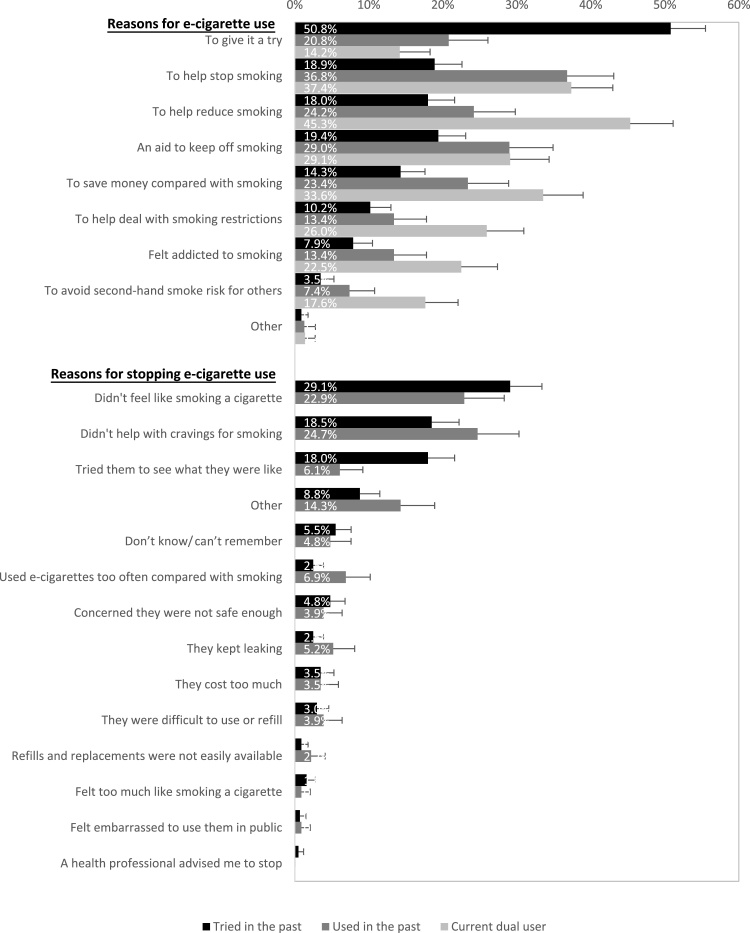
Reasons for using (*N* = 953) and stop using (*N* = 664) e-cigarettes by smokers’ e-cigarette use status.

Smokers who had ever tried e-cigarettes could choose multiple responses for use and those who had tried or used in the past were asked for their reasons not to continue e-cigarette use (Fig. 1).

### 2.3 Procedures

The Smokefree Britain survey is commissioned annually by the charity Action on Smoking and Health (ASH, 2016b) and uses a panel of around 816,000 UK adults (aged 18 + ) maintained by the market research company YouGov Plc. Panel members were emailed an invitation to participate without information on survey content. Members who agreed were allocated in line with quotas for age and gender (interlocked), social grade, newspaper readership, ethnicity, and region to achieve a good representation of the adult GB population. Panel members consent to completing surveys for a modest financial incentive, and additional ethical approval was not sought due to this pre-existing consent. All recodes and analyses for the present manuscript were run by the authors using data collected by YouGov (YouGov, 2016). Fieldwork was undertaken 2nd to 23rd March 2016.

### 2.4 Analysis

Descriptive statistics summarised sociodemographic (age, gender, social grade) and smoking (HSI, MTSS) characteristics, and type of first e-cigarette used. Differences by e-cigarette use status were assessed using chi-square statistics. For statistically significant and larger than 2 × 2 contingency tables, adjusted residuals >±2.58 identified cells contributing to differences between groups (Sharpe, 2015).

For research question 1, multivariable logistic regressions assessed the associations between e‐cigarette use status, gender, age, social grade, HSI, and first e-cigarette type and MTSS.

To address research question 2, proportions endorsing reasons were presented with 95% confidence intervals. A multinomial logistic regression compared past triers’ and current dual users’ reasons for use with past users’ reasons, adjusting for gender, age, social grade, HSI, MTSS, and type of first e-cigarette.

## 3 Results

### 3.1 Characteristics by e‐cigarette use status

Smokers who had never tried e‐cigarettes, tried in the past, used in the past, and dual users did not differ by gender or social grade, but by age (Table 1). Past users had the largest proportion of high dependence, and past triers were less likely to have first tried tank types than current dual users (Table 1).

### 3.2 E-cigarette use and motivation to stop smoking

Age, dependence, and e-cigarette use status were associated with motivation to stop smoking in the next three months (Table A1). Thirty‐five to 44‐year‐old smokers were more likely to be motivated to stop smoking than 18–24‐year‐olds (AOR = 2.68, 95% CI: 1.18–6.10). Highly dependent smokers were less likely to be motivated than less dependent smokers (AOR = 0.39, 95% CI: 0.21–0.70). Current dual users were more likely to be motivated to stop smoking than past users (AOR = 1.95, 95% CI: 1.10–3.46); never users’ (AOR = 0.89, 95% CI: 0.50–1.57) and past triers’ motivation (AOR = 1.20, 95% CI: 0.68–2.13) was similar to past users’. Post-hoc analysis showed no association between e-cigarette type and motivation to stop smoking (Table A2).

### 3.3 Reasons for and characteristics associated with using and discontinuing e-cigarettes

The three most common reasons for using e-cigarettes were to give it a try, to help stop smoking, and to reduce the amount of tobacco smoked (Fig. 1). Past triers’ single top reason was ‘to give it a try’, while past users had mostly used e-cigarettes as an aid to stop, keep off, or reduce smoking. Current dual users used e-cigarettes to help reduce or stop smoking, and to save money (Fig. 1).

Compared with past users, past triers were less likely to endorse ‘to help stop smoking’ (AOR = 0.46, 95% CI: 0.33–0.73) and more likely to endorse ‘to give it a try' (AOR = 2.99, 95% CI: 1.99–4.50), more likely to be female (AOR = 1.44, 95% CI: 1.01–2.06) and motivated to stop smoking (AOR = 1.84, 95% CI: 1.00–3.38), and less likely to be 55 or older (AOR = 0.44, 95% CI: 0.22–0.85). Current dual users were more likely to endorse smoking reduction (AOR = 2.40, 95% CI: 1.59–3.64) and overcoming smoking restrictions (AOR = 2.03, 95% CI: 1.22–3.38), were less dependent (AOR = 0.54, 95% CI: 0.35-0.86), and more motivated to stop smoking (AOR = 2.44, 95% CI: 1.33-4.50) than past users (Table A3).

Smokers mostly stopped using e-cigarettes because it did not feel like smoking a tobacco cigarette, did not help with cravings, and because they had tried them just to see what they were like (Fig. 1); the latter was endorsed more often by those who had tried rather than used in the past.

## 4 Discussion

Smokers differed in motivation to stop smoking according to their e‐cigarette use experience. Dual users of tobacco and e-cigarettes had higher motivation to stop smoking than past users who, in turn, had similar levels of motivation as smokers who had never used or had tried e-cigarettes. Dual users used e-cigarettes to help reduce smoking and to deal with smoking restrictions, while smokers who had tried e-cigarettes for curiosity reasons were unlikely to have continued use. Smokers had mostly discontinued vaping because it did not feel like regular cigarettes or did not help with cravings, and they were more dependent than current dual users.

The cross-sectional study design predisposes the findings to certain limitations. No causal statements can be made about the observed associations. Data were self-reported at one time‐point, therefore answers could have been affected by recall bias, and current motivation to stop smoking may have been less relevant for past e-cigarette use. The analysed sample did not include past smokers who may have stopped smoking while vaping, nor could we verify that those who tried or used e-cigarettes in the past were smokers at that time.

Our finding that currently vaping smokers were more motivated to stop smoking than others supports evidence from the US (Nayak et al., 2016, Rutten et al., 2015). Smokers’ reasons for e-cigarette use and discontinuation were similar to previous findings (Biener and Hargraves, 2015, Biener et al., 2015, McNeill et al., 2015, Pepper et al., 2014, Rutten et al., 2015), and our findings support a differentiation between goal and non-goal-oriented reasons for use, with the latter less likely to lead to continued use (Pepper et al., 2014).

Smokers who had stopped vaping were more dependent than current dual users. To note, dependence was measured using the Heaviness of Smoking index, which might be lower among dual users who tend to reduce the number of cigarettes (Brose et al., 2015, Manzoli et al., 2016, McRobbie et al., 2014). The main reasons for discontinuation suggest that more dependent smokers may find vaping not sufficient, thus increasing their risk of stopping use but continuing to smoke. Our findings may suggest that dual use represents a transient phase of heightened motivation to stop smoking, thus smokers using e-cigarettes should be encouraged to stop smoking completely and supported to reduce cravings.

Future longitudinal research could appraise the temporality of the association between e-cigarette use and motivation to stop smoking. Furthermore, focussing on highly dependent smokers’ experiences of using e-cigarettes could clarify risks for reverting from dual use to smoking only.

## 5 Conclusions

Among smokers, having used e-cigarettes out of curiosity is associated with discontinued use, while ongoing dual use is associated with aiming to reduce smoking and overcome smoking restrictions, heightened motivation to stop smoking, and lower dependence. Smokers discontinue using e-cigarettes mostly because they lack resemblance to cigarettes and do not reduce cravings, or because smokers just wanted to try them.

## Conflict of interest

No conflict declared.

## Role of funding source

This study was supported by a number of sources. LB and ES are funded by a Cancer Research UK (CRUK)/BUPA Foundation Cancer Prevention Fellowship (C52999/A19748); some of the work on this study was funded by (C25586/A19540). LB and AMcN are members of the UK Centre for Tobacco and Alcohol Studies, a UK Clinical Research Collaboration Public Health Research: Centre of Excellence. Funding from the Medical Research Council, British Heart Foundation, Cancer Research UK, Economic and Social Research Council, and the National Institute for Health Research under the auspices of the UK Clinical Research Collaboration is gratefully acknowledged (MR/K023195/1). DA is employed by Action on Smoking & Health which receives funding from CRUK, British Heart Foundation, and the Department of Health.

## Contributors

All authors contributed to the design of the study, and interpretation of the data. ES led the data analysis for the manuscript and wrote the first draft. All authors contributed to the amendment of the manuscript and approved its final version. DA commissioned YouGov to conduct the survey. This study uses data which YouGov collected and shared with the authors who conducted all analyses reported here.

## Figures and Tables

**Table 1:** Smokers’ sociodemographic, smoking, and e-cigarette use characteristics by their e-cigarette use experience. *N* = 1489.

	Never e-cigarette users *n* = 536 (36%)	Tried in the past *n* = 433 (29.1%)	Used in the past *n* = 231 (15.5%)	Current dual users *n* = 289 (19.4%)	Comparison statistic
Female% (*n*)	54.1 (290)	56.6 (245)	53.7 (124)	51.2 (148)	χ^2^(3) = 2.06, *p* = 0.56

Age group% (*n*)					
18–24	9 (48)	14.5 (63)*	6.5 (15)	3.1 (9)*	**χ^2^(12)** **=** **51.63, *p <*** **0.001**
25–34	7.6 (41)	12 (52)*	6.1 (14)	8.3 (24)	
35–44	14.4 (77)	17.1 (74)	16 (37)	14.2 (41)	
45–54	20.3 (109)	19.9 (86)	18.6 (43)	21.8 (63)	
55+	48.7 (261)	36.5 (158)*	52.8 (122)	52.6 (152)	

ABC1 social grade% (*n*)	53.7 (288)	52.7 (228)	48.5 (112)	51.9 (150)	χ^2^(3) = 1.83, *p* = 0.61

MTSS% (n)					
I don’t want to ever stop smoking	10.1 (54)*	6.7 (29)	4.8 (11)	4.5 (13)	**χ^2^(21)** **=** **54.28, *p <*** **0.001**
I think I should stop smoking but don’t really want to	40.3 (216)	39.3 (170)	42 (97)	29.4 (85)*	
I want to stop smoking but haven’t thought about when	12.3 (66)	11.8 (51)	9.5 (22)	7.3 (21)	
I want to stop smoking and hope to soon	14.6 (78)	15.7 (68)	14.3 (33)	21.1 (61)*	
I REALLY want to stop smoking but I don’t know when I will	11.9 (64)	13.4 (58)	19 (44)	17 (49)	
I REALLY want to stop smoking and intend to in the next 3 months	3.7 (20)	5.3 (23)	4.8 (11)	8.7 (25)*	
I REALLY want to stop smoking and intend to in the next month	4.1 (22)	5.1 (22)	3.5 (8)	7.3 (21)	
Don’t know	3 (16)	2.8 (12)	2.2 (5)	4.8 (14)	

Motivation to stop in next 3 months% (*n*)	7.8 (42)	10.4 (45)	8.2 (19)	15.9 (46)*	**χ^2^(3)** **=** **14.57, *p*** **=** **0.002**

High dependence (HSI ≥ 4) % (*n*)	16.6 (89)	18.7 (81)	27.3 (63)*	17 (49)	**χ^2^(3)** **=** **13.11, *p*** **=** **0.004**

Type of the first e-cigarette used% (*n*)					
Disposable e-cigarette		19.9 (86)	17.3 (40)	16.6 (48)	**χ^2^(6)** **=** **38.29, *p <*** **0.001**
Rechargeable e-cigarette		39.5 (171)	43.3 (100)	38.8 (112)	
Tank e-cigarette		30.3 (131)*	36.4 (84)	43.3 (125)*	
Don’t know/Other		10.4 (45)*	3.0 (7)	1.4 (4)*	

*Note*. MTSS: Motivation to Stop Smoking; HSI: Heaviness of Smoking Index; M: mean; SD: standard deviation.

* Cells associated with adjusted residuals greater than ±2.58 (α = 0.01).

## References

[bib0005] ASH (2016). Use of Electronic Cigarettes (vapourisers) Among Adults in Great Britain. http://ash.org.uk/information-and-resources/fact-sheets/use-of-electronic-cigarettes-vapourisers-among-adults-in-great-britain/.

[bib0010] ASH (2016). About Action on Smoking and Health [WWW Document]. http://www.ash.org.uk/about-ash.

[bib0015] Amato M.S., Boyle R.G., Levy D. (2016). How to define e-cigarette prevalence? Finding clues in the use frequency distribution. Tob. Control.

[bib0020] Beard E., McNeill A., Aveyard P., Fidler J., Michie S., West R. (2013). Association between use of nicotine replacement therapy for harm reduction and smoking cessation: a prospective study of English smokers. Tob. Control.

[bib0025] Biener L., Hargraves J.L. (2015). A longitudinal study of electronic cigarette use among a population-based sample of adult smokers: association with smoking cessation and motivation to quit. Nicotine Tob. Res..

[bib0030] Biener L., Song E., Sutfin E., Spangler J., Wolfson M. (2015). Electronic cigarette trial and use among young adults: reasons for trial and cessation of vaping. Int. J. Environ. Res. Public Health.

[bib0035] Brose L.S., Hitchman S.C., Brown J., West R., McNeill A. (2015). Is the use of electronic cigarettes while smoking associated with smoking cessation attempts, cessation and reduced cigarette consumption? A survey with a 1-year follow-up. Addiction.

[bib0040] Diaz F.J., Jané M., Saltó E., Pardell H., Salleras L., Pinet C., de Leon J. (2005). A brief measure of high nicotine dependence for busy clinicians and large epidemiological surveys. Aust. N. Z. J. Psychiatry.

[bib0045] Grana R., Benowitz N., Glantz S.A. (2014). E-cigarettes: a scientific review. Circulation.

[bib0050] Heatherton T.F., Kozlowski L.T., Frecker R.C., Rickert W., Robinson J. (1989). Measuring the heaviness of smoking: using self-reported time to the first cigarette of the day and number of cigarettes smoked per day. Br. J. Addict..

[bib0055] Hitchman S.C., Brose L.S., Brown J., Robson D., McNeill a. (2015). Associations between e-cigarette type, frequency of use, and quitting smoking: findings from a longitudinal online panel survey in Great Britain. Nicotine Tob. Res. 1.

[bib0060] Hummel K., Brown J., Willemsen M.C., West R., Kotz D. (2016). External validation of the motivation to stop scale (MTSS): findings from the international tobacco control (ITC) Netherlands survey. Eur. J. Public Health.

[bib0065] Kotz D., Brown J., West R. (2013). Predictive validity of the Motivation To Stop Scale (MTSS): A single-item measure of motivation to stop smoking. Drug Alcohol Depend..

[bib0070] MHRA (2013). Licensing Procedure for Electronic Cigarettes and Other Nicotine Containing Products (NCPs) as Medicines [WWW Document]. http://www.mhra.gov.uk/home/groups/comms-ic/documents/websiteresources/con454361.pdf.

[bib0075] MHRA (2015). Public Assessment Report for e-Voke 10 Mg and 15 Mg Electronic Inhaler [WWW Document]. http://www.mhra.gov.uk/home/groups/par/documents/websiteresources/con616843.pdf.

[bib0080] Manzoli L., Flacco M.E., Ferrante M., La Vecchia C., Siliquini R., Ricciardi W., Marzuillo C., Villari P., Fiore M. (2016). Cohort study of electronic cigarette use: effectiveness and safety at 24 months. Tob. Control.

[bib0085] McNeill A., Brose L.S., Calder R., Hitchman S.C., Hajek P.H.M. (2015). E-cigarettes: An Evidence Update A Report Commissioned by Public Health England.

[bib0090] McRobbie H., Bullen C., Hartmann-Boyce J., Hajek P. (2014). Electronic cigarettes for smoking cessation and reduction. Cochrane database Syst. Rev..

[bib0095] National Institute for Health and Care Excellence (2013). Smoking: Harm Reduction Guidline PH45.

[bib0100] National Readership Survey (2016). Social Grade [WWW Document]. http://www.nrs.co.uk/nrs-print/lifestyle-and-classification-data/social-grade/.

[bib0105] Nayak P., Pechacek T.F., Weaver S.R., Eriksen M.P. (2016). Electronic nicotine delivery system dual use and intention to quit smoking: will the socioeconomic gap in smoking get greater?. Addict. Behav..

[bib0110] Pepper J.K., Ribisl K.M., Emery S.L., Brewer N.T. (2014). Reasons for starting and stopping electronic cigarette use. Int. J. Environ. Res. Public Health.

[bib0115] Royal College of Physicians (2016). Nicotine Without Smoke: Tobacco Harm Reduction.

[bib0120] Rutten L.J.F., Blake K.D., Agunwamba A.A., Grana R.A., Wilson P.M., Ebbert J.O., Okamoto J., Leischow S.J. (2015). Use of e-cigarettes among current smokers: associations among reasons for use, quit intentions, and current tobacco use. Nicotine Tob. Res..

[bib0125] Sharpe D. (2015). Your chi-Square test is statistically significant: now what?. Pract. Assess. Res. Eval..

[bib0130] Stead L.F., Lancaster T., Stead L.F. (2007). Interventions to reduce harm from continued tobacco use. Cochrane Database of Systematic Reviews.

[bib0135] Stead L.F., Perera R., Bullen C., Mant D., Lancaster T. (2008). Nicotine replacement therapy for smoking cessation. Cochrane database Syst. Rev..

[bib0140] Wagener T.L., Meier E., Hale J.J., Oliver E.R., Warner M.L., Driskill L.M., Gillaspy S.R., Siegel M.B., Foster S. (2014). Pilot investigation of changes in readiness and confidence to quit smoking after E-cigarette experimentation and 1 week of use. Nicotine Tob. Res..

[bib0145] West R., Brown J. (2015). Electronic Cigarette Use for Quitting Smoking in England: 2015.

[bib0150] West R., Brown J., Beard E. (2016). Trends in Electronic Cigarette Use in England.

[bib0155] YouGov (2016). YouGov | Panel Methodology [WWW Document]. https://yougov.co.uk/about/panel-methodology/.

