# Supplementary material for: What factors are associated with current smokers using or stopping e-cigarette use?
Source: Drug Alcohol Depend. 2017 Apr 1;173:139–43. doi: 10.1016/j.drugalcdep.2017.01.002 (PMC5380653; doi:10.1016/j.drugalcdep.2017.01.002)
Supplement: Supplementary file 1 [file mmc1.docx]

**Supplementary Material for the Article:**

What factors are associated with current smokers using or stopping e-cigarette use?

Erikas Simonavicius^a^, Ann McNeill^a^, Deborah Arnott^b^, Leonie S Brose^a^

^a^ Department of Addictions, Institute of Psychiatry, Psychology and Neuroscience, King’s College London, London, United Kingdom

^b^ Action on Smoking and Health, London, United Kingdom

Corresponding author: Erikas Simonavicius, Department of Addictions, Institute of Psychiatry, Psychology and Neuroscience, King’s College London, 4 Windsor Walk, London SE5 8BB, United Kingdom. E-mail: [erikas.simonavicius@kcl.ac.uk](mailto:erikas.simonavicius@kcl.ac.uk)

**This material supplements, but does not replace, the peer-reviewed paper in**

***Drug and Alcohol Dependence*.**

**Table A1**

Logistic regression predicting motivation to stop smoking in next 3 months; *N* = 1,489.

|  |  |  | **Bivariate** | | | **Multivariable** | | |
| --- | --- | --- | --- | --- | --- | --- | --- | --- |
| **Variable** | ***N*** | **% intending to stop**  **in next 3 months** | **Odds Ratio** | **95% confidence intervals** | ***p*** | **Odds Ratio** | **95% confidence intervals** | ***p*** |
| Gender |  |  |  |  |  |  |  |  |
| Male | 682 | 10.7 | ref |  |  | ref |  |  |
| Female | 807 | 9.8 | 0.91 | 0.65 - 1.27 | .56 | 0.95 | 0.67 - 1.34 | .77 |
|  |  |  |  |  |  |  |  |  |
| Age |  |  |  |  |  |  |  |  |
| 18-24 | 135 | 5.9 | ref |  |  | ref |  |  |
| 25-34 | 131 | 14.5 | **2.69** | **1.13 - 6.39** | **.025** | 2.40 | 1.00 - 5.79 | .051 |
| 35-44 | 229 | 14.8 | **2.77** | **1.24 - 6.17** | **.013** | **2.68** | **1.18 - 6.10** | **.019** |
| 45-54 | 301 | 11 | 1.95 | 0.88 - 4.35 | .101 | 1.90 | 0.84 - 4.31 | .125 |
| 55+ | 693 | 8.4 | 1.45 | 0.68 - 3.11 | .34 | 1.45 | 0.66 - 3.18 | .35 |
|  |  |  |  |  |  |  |  |  |
| Social grade |  |  |  |  |  |  |  |  |
| ABC1 | 778 | 10.9 | ref |  |  | ref |  |  |
| C2DE | 711 | 9.4 | 0.85 | 0.61 - 1.19 | .34 | 0.95 | 0.67 - 1.34 | .75 |
|  |  |  |  |  |  |  |  |  |
| HSI |  |  |  |  |  |  |  |  |
| Low dependence  (HSI < 4) | 1,207 | 11.5 | ref |  |  | ref |  |  |
| High dependence  (HSI ≥ 4) | 282 | 4.6 | **0.37** | **0.21 - 0.67** | **.001** | **0.39** | **0.21 - 0.70** | **.002** |
|  |  |  |  |  |  |  |  |  |
| E-cigarette use |  |  |  |  |  |  |  |  |
| Used in the past | 231 | 8.2 | ref |  |  | ref |  |  |
| Never user | 536 | 7.8 | 0.95 | 0.54 - 1.67 | .86 | 0.89 | 0.50 - 1.57 | .68 |
| Tried in the past | 433 | 10.4 | 1.29 | 0.74 - 2.27 | .37 | 1.20 | 0.68 - 2.13 | .53 |
| Current dual user | 289 | 15.9 | **2.11** | **1.20 - 3.72** | **.010** | **1.95** | **1.10 - 3.46** | **.022** |

Note. HSI: Heaviness of Smoking Index; ref: reference category. R^2^ = .026 (Cox & Snell), .053 (Nagelkerke). Model χ^2^(10) = 38.79, *p* < .001.

**Table A2**

Logistic regression predicting motivation to stop smoking in next 3 months among smokers who had tried e-cigarettes; *N* = 953.

|  |  |  | **Bivariate** | | | **Multivariable** | | |
| --- | --- | --- | --- | --- | --- | --- | --- | --- |
| **Variable** | ***N*** | **% intending to stop**  **in next 3 months** | **Odds Ratio** | **95% confidence intervals** | ***p*** | **Odds Ratio** | **95% confidence intervals** | ***p*** |
| Gender |  |  |  |  |  |  |  |  |
| Male | 436 | 13.1 | ref |  |  | ref |  |  |
| Female | 517 | 10.3 | 0.76 | 0.51 - 1.13 | .76 | 0.81 | 0.54 - 1.21 | .31 |
|  |  |  |  |  |  |  |  |  |
| Age |  |  |  |  |  |  |  |  |
| 18-24 | 87 | 4.6 | ref |  |  | ref |  |  |
| 25-34 | 90 | 15.6 | **3.82** | **1.21-12.12** | **.023** | **3.34** | **1.03-10.79** | **.044** |
| 35-44 | 152 | 17.1 | **4.28** | **1.44-12.72** | **.009** | **3.98** | **1.30-12.12** | **.015** |
| 45-54 | 192 | 13.0 | **3.11** | **1.05 - 9.22** | **.041** | 2.79 | 0.92 - 8.47 | .070 |
| 55+ | 432 | 9.5 | 2.18 | 0.76 - 6.24 | .148 | 1.98 | 0.67 - 5.84 | .22 |
|  |  |  |  |  |  |  |  |  |
| Social grade |  |  |  |  |  |  |  |  |
| ABC1 | 490 | 12.4 | ref |  |  | ref |  |  |
| C2DE | 463 | 10.6 | 0.83 | 0.56 - 1.24 | .37 | 0.96 | 0.63 - 1.45 | .85 |
|  |  |  |  |  |  |  |  |  |
| HSI |  |  |  |  |  |  |  |  |
| Low dependence  (HSI < 4) | 760 | 13.0 | ref |  |  | ref |  |  |
| High dependence  (HSI ≥ 4) | 193 | 5.7 | **0.40** | **0.21 - 0.77** | **.006** | **0.45** | **0.23 - 0.86** | **.016** |
|  |  |  |  |  |  |  |  |  |
| E-cigarette use |  |  |  |  |  |  |  |  |
| Used in the past | 231 | 8.2 | ref |  |  | ref |  |  |
| Tried in the past | 433 | 10.4 | 1.29 | 0.74 - 2.27 | .37 | 1.27 | 0.71 - 2.27 | .42 |
| Current dual user | 289 | 15.9 | **2.11** | **1.20 - 3.72** | **.010** | **2.00** | **1.12 - 3.56** | **.019** |
|  |  |  |  |  |  |  |  |  |
| Type of the first e-cigarette used |  |  |  |  |  |  |  |  |
| Disposable e-cigarette | 174 | 9.8 | ref |  |  | ref |  |  |
| Rechargeable e-cigarette | 383 | 13.3 | 1.42 | 0.79 - 2.54 | .24 | 1.50 | 0.83 - 2.71 | .185 |
| Tank e-cigarette | 340 | 10.9 | 1.13 | 0.62 - 2.07 | .70 | 1.12 | 0.60 - 2.09 | .72 |
| Don’t know/ Other | 56 | 8.9 | 0.91 | 0.32 - 2.58 | .85 | 0.99 | 0.34 - 2.91 | .99 |

Note. HSI: Heaviness of Smoking Index; ref: reference category. R^2^ = .033 (Cox & Snell), .064 (Nagelkerke). Model χ^2^(12) = 31.84, *p* = .001.

**Table A3**

Multinomial logistic regression appraising associations with past e-cigarette trial and current dual use (compared with past e-cigarette users); *N* = 953.

| **Variable** | **Used in the past** | **Tried in the past vs.**  **Used in the past (ref)** | | | **Current dual users vs.**  **Used in the past (ref)** | | |
| --- | --- | --- | --- | --- | --- | --- | --- |
|  | **Odds ratio** | **Odds Ratio** | **95% confidence intervals** | ***p*** | **Odds Ratio** | **95% confidence intervals** | ***p*** |
| Reasons for using e-cigarettes |  |  |  |  |  |  |  |
| To help stop smoking | 1.00 | **0.49** | **0.33 - 0.73** | **< .001** | 1.05 | 0.70 - 1.57 | .82 |
| To help reduce smoking | 1.00 | 0.89 | 0.58 - 1.35 | .57 | **2.40** | **1.59 - 3.64** | **< .001** |
| An aid to keep off smoking | 1.00 | 0.78 | 0.52 - 1.18 | .24 | 0.82 | 0.54 - 1.26 | .37 |
| To help deal with smoking restrictions | 1.00 | 0.83 | 0.48 - 1.41 | .49 | **2.03** | **1.22 - 3.38** | **.006** |
| To avoid second-hand smoke risk for others | 1.00 | 0.64 | 0.30 - 1.38 | .26 | 1.69 | 0.89 - 3.20 | .109 |
| To save money compared with smoking | 1.00 | 0.76 | 0.48 - 1.19 | .23 | 1.37 | 0.88 - 2.12 | .161 |
| Felt addicted to smoking | 1.00 | 0.82 | 0.47 - 1.40 | .82 | 1.52 | 0.91 - 2.51 | .107 |
| To give it a try | 1.00 | **2.99** | **1.99 - 4.50** | **< .001** | 0.69 | 0.42 - 1.13 | .137 |
| Other | 1.00 | 0.71 | 0.14 - 3.50 | .67 | 1.43 | 0.30 - 6.78 | .65 |
|  |  |  |  |  |  |  |  |
| Gender |  |  |  |  |  |  |  |
| Male |  | ref |  |  | ref |  |  |
| Female | 1.00 | **1.44** | **1.01 - 2.06** | **.043** | 0.90 | 0.62 - 1.31 | .60 |
|  |  |  |  |  |  |  |  |
| Age |  |  |  |  |  |  |  |
| 18-24 |  | ref |  |  | ref |  |  |
| 25-34 | 1.00 | 0.97 | 0.41 - 2.32 | .95 | 2.84 | 0.95 - 8.48 | .061 |
| 35-44 | 1.00 | 0.59 | 0.28 - 1.24 | .166 | 1.51 | 0.57 - 4.04 | .41 |
| 45-54 | 1.00 | 0.74 | 0.36 - 1.53 | .41 | 1.71 | 0.65 - 4.45 | .28 |
| 55+ | 1.00 | **0.44** | **0.22 - 0.85** | **.014** | 1.79 | 0.73 - 4.40 | .21 |
|  |  |  |  |  |  |  |  |
| Social grade |  |  |  |  |  |  |  |
| ABC1 |  | ref |  |  | ref |  |  |
| C2DE | 1.00 | 0.79 | 0.56 - 1.13 | .196 | 0.90 | 0.62 - 1.31 | .58 |
|  |  |  |  |  |  |  |  |
| MTSS |  |  |  |  |  |  |  |
| No motivation to stop in next 3 months |  | ref |  |  | ref |  |  |
| Motivation to stop in next 3 months | 1.00 | **1.84** | **1.00 - 3.38** | **.050** | **2.44** | **1.33 - 4.50** | **.004** |
|  |  |  |  |  |  |  |  |
| HSI |  |  |  |  |  |  |  |
| Low dependence |  | ref |  |  | ref |  |  |
| High dependence | 1.00 | 0.80 | 0.53 - 1.21 | .30 | **0.54** | **0.35 - 0.86** | **.009** |
|  |  |  |  |  |  |  |  |
| Type of the first e-cigarette used |  |  |  |  |  |  |  |
| Disposable e-cigarette |  | ref |  |  | ref |  |  |
| Rechargeable e-cigarette | 1.00 | 0.98 | 0.61 - 1.59 | .93 | 0.76 | 0.45 - 1.30 | .32 |
| Tank e-cigarette | 1.00 | 0.82 | 0.50 - 1.35 | .43 | 1.27 | 0.75 - 2.17 | .37 |
| Don’t know/ Other | 1.00 | 2.48 | 0.98 - 6.27 | .055 | 0.58 | 0.15 - 2.17 | .58 |

Note. Note. MTSS: Motivation to Stop Smoking; HSI: Heaviness of Smoking Index, ref: reference category.

Pseudo R^2^ = .289 (Cox & Snell), .328 (Nagelkerke), .160 (McFadden). Model χ^2^(1372) = 1490.87, *p* = .013.
